# Factors affecting compliance with national accreditation essential safety standards in the Kingdom of Saudi Arabia

**DOI:** 10.1038/s41598-022-11617-7

**Published:** 2022-05-09

**Authors:** Arwa Althumairi, Amal Alzahrani, Turki Alanzi, Salem Al Wahabi, Summaya Alrowaie, Afnan Aljaffary, Duaa Aljabri

**Affiliations:** 1 Department of Health Information Management and Technology, College of Public Health, Imam Abdulrahman Bin Faisal University, P. O. Box 2954, Dammam, 6603-34211 Saudi Arabia; 2 Central Board for Accreditation of Healthcare Institutions-CBAHI, Riyadh, Saudi Arabia

**Keywords:** Health care, Health occupations, Medical research

## Abstract

Accreditation is a widespread culture internationally and nationally. The effectiveness of compliance with accreditation standards was positively correlated with health care settings’ performance in multiple aspects: leadership, professional performance, patient safety and organizational culture. There is limited knowledge of the national compliance rate with accreditation standards. Therefore, it is important to assess the hospital compliance with accreditation rate in the Kingdom Saudi Arabia (KSA) and its related factors. This paper presents a quantitative cross-sectional study. Data were extracted from the annual Essential Safety Requirement (ESR) survey database from the Central Board for Accreditation of Health care Institutions (CBAHI) research center during the period 2016 to 2018. Hospitals that started their operation after the first ESR survey round in 2016 or shut down during the study period were excluded. The hospital scoring was on a scale of 0 to 100 and classified as follows: score 2 if the hospital satisfactory compliance (Fully Met) was ≥ 80% and score 1 if particular compliance (Partially Met) was ≥ 50% to < 80%. Then, a score of 0 indicated insufficient compliance (Not Met) when < 50% and a score of not applicable (NA) if the standard does not apply to the hospital. A total of 437 hospitals were surveyed in 20 regions in the KSA and had an overall compliance rate on average that was higher among private hospitals than among public hospitals (77% vs. 66%). Overall, private hospitals had a significantly better compliance rate than public hospitals (mean rate = 84% vs. 68%, respectively, P = 0.019). Large hospitals had more compliance with some standards than smaller hospitals. After adjusting for the year of the survey report, the private hospital type was more compliant than the public hospital. This study supports mandatory accreditation programs for both public and private health sectors, with increased monitoring by the concerned parties (i.e., CBAHI and the Ministry of Health). The authors encourage the application of accreditation for specialized and independent health services.

## Introduction

Accreditation formally started in the United States with the formation of The Joint Commission on accreditation of health care organizations in 1951. This model was exported to Canada and Australia in the 1960s and 1970s and reached Europe in the 1980s. Accreditation programs spread all over the world in the 1990s^1^. In the Kingdom of Saudi Arabia (KSA), the national accreditation body for health care institutions is the Central Board for Accreditation of Health care Institutions “CBAHI”, which started as a voluntary program in 2005 and then became mandatory in 2014. CBAHI is the only national agency authorized to grant accreditation certificates to all governmental and private health care facilities. Their principal function is to set the health care quality and patient safety standards against which all health care facilities are evaluated for compliance.

In 2016, the National Transformation Program for Ministry of Health established the Essential Safety Requirements (ESR) standards initiative, which became a mandatory prerequisite for accreditation generated by CBAHI as the minimum required for hospitals to be eligible for accreditations^2^. Thus, any hospital in the KSA shall not be able to receive national accreditation from CBAHI without producing evidence on its (85% and above) compliance with ESR standards^3^.

The rate of adopting mandatory accreditation standards varies among different types of private health care providers^4^. These were due to three factors: concerns of first, financial consequences, then, knowledge and awareness of accreditation, and finally, professional support to implement the standards^4^. A study focused on the Hospital-Acquired Condition Reduction Program found that 79.3% of respondents believed that the standard rate would be improved if it was tailored to the type and size of hospitals, even though they highly agreed (77.2%) that the size and type of hospitals would not impede the application of standards and that the standards could be implemented anyhow^5^.

It is important to understand the status of compliance with accreditation guidelines to provide a baseline of the quality level of health care delivery in the current study area compared to different health care delivery provided worldwide. This study aims to identify the factors that influence the compliance rate for ESR overall and for each ESR. Additionally, we identified the factors that influence the compliance rate by year.

## Methods

### Study design

A descriptive retrospective study design.

### Data source

Data were extracted from the annual ESR survey database. All hospitals of the KSA that underwent the ESR survey process from the beginning of its first survey round in 2016 until its third round in 2018 were included in the analysis. Hospitals that started their operation after the first ESR survey round in 2016 or shut down during the study period were excluded. A list of ESRs is identified in Table 1.

**Table 1:** List of essential safety requirement codes and definitions.

Standard code	Code definition
HR.5	The hospital has a process for proper credentialing of staff members licensed to provide patient care
MS.7	Medical staff members have current delineated clinical privileges
PC.25	Policies and procedures guide the handling, use, and administrations of blood and blood products
PC.26	Patients at risk for developing venous thromboembolism are identified and managed
QM.17	The hospital has a process to ensure correct identification of patients
QM.18	The hospital had a process to prevent wrong patient, wrong site, and wrong surgery/procedure
AN.2	Anesthesia staff members have the appropriate qualifications
AN.15	Qualified staff perform moderate and deep sedation/analgesia
IPC.4	There is a designated multidisciplinary committee that provides oversight of the infection prevention and control program
IPC.15	Facility design and available supplies support isolation practices
MM.5	The hospital has a system for the safety of high-alert medications
MM.6	The hospital has a system for safety of look-alike and sound-alike (LASA) medications
MM.41	The hospital has a process for monitoring, identifying, and reporting significant medication errors, including near misses, hazardous conditions, and at-risk behaviors that have the potential to cause patient harm. potential to cause patient harm
LB.51	The blood bank develops a process to prevent disease transmission by blood/platelet transfusion
FMS.9	The hospital ensures that all occupants are safe from radiation hazards
FMS.32	The hospital ensures proper maintenance of the medical gas system
FMS.21	The hospital had an effective fire alarm system
FMS.22	The hospital has a fire suppression system available in the required area(s)
FMS.23	There are fire exits that are properly located in the hospital
FMS.24	The hospital and its occupants are safe from fire and smoke

### Data collection methods

All data were collected from secondary data extracted from the ESR annual report database in the CBAHI Center for all hospitals across the KSA that received accreditation from 2016 to 2018.

#### Instruments

The ESR survey at the CBAHI organization was collected based on a one-day on-site survey visit carried out by one or two surveyors. Depending on the number of the hospital-trained ESR surveyors, the ESR standards were derived from several activities, including observations, interviews, document review and unit visits. The ESR surveyors self-administered a score for each standard through a postal survey system on a CBAHI portal. Each standard was scored according to a scale of 100 points, and then an automatic calculation of the average (arithmetic mean) score of all applicable substandards was estimated.

The hospital passed if the overall score was ≥ 85%. (CBAHI, CBAHI Standards, 2016) Following the completion of the survey process, an integrated database containing the overall scores and individual scores for each standard for each hospital was created. After contacting the CBAHI organization research center and obtaining official permission, data from the annual ESR survey database from 2016 to 2018 were received.

#### Study variables

The dependent variables are the average compliance rate overall and for each ESR standard. The independent variable was the year of the surveys, which was defined as a categorical variable. Additional variables included hospital type, which was defined as a binary variable (public and private); hospital bed capacity, which was defined as a binary variable (≤ 200 beds, > 200 beds); and the regions where the hospitals were located (Eastern, Western, Central, Northern, and Southern).

#### Procedure and timeline

Using secondary data collection, researchers retrieved data from the CBAHI database for more than 470 hospitals all over the KSA and focused on hospital types, regions, sectors and bed capacities; the scores for all hospitals participating in the ESR surveys.

#### Analysis

All analyses were conducted by using Statistical Package for the Social Sciences (SPSS) v 25, Armonk, NY. After the distribution was confirmed to be normal, the following analyses were adopted.

A descriptive analysis was used to assess the prevalence of hospital inclusion by hospital type (privet or public), bed capacity, year, and region. A bivariate analysis was used to confirm that the dependent variable (compliance rate for each standard and total) was normally distributed. An independent *t* test was used to compare the means of the compliance rate compared for the two independent categorical groups for both variables of hospital type and hospital size “bed capacity”, while a one-way ANOVA test was used to compare the mean compliance rate for the three independent groups of years (2016, 2017, and 2018). A 95% confidence interval was used to assess the significant finding. Finally, multivariable analysis was used to test the hypothesis that private hospitals had a higher compliance rate than public hospitals. Linear regression analysis was used to make predictions and model the relationship between the compliance rate and the explanatory variables (hospital type and size), and P < 0.05 was considered statistically significant.

### Ethics approval and consent to participate

Ethical approval was given by Imam Abdurrahman bin Faisal University to conduct this research under ethical approval number IRB-PGS-2018-03-295, approved 19/12/2018. The data are based on secondary data, where the host of the data source has the right to use the data for research purposes.

### Consent for publication

The authors agreed to share the research material for publication purposes.

## Results

### Univariate analysis

A total of 437 hospitals were included in the survey. Table 2 presents the characteristics of the hospitals, along with their average compliance rate with ESR standards. A clear difference was seen in the improvement of the average compliance rate from 2016 to 2018 on all levels. Overall, the compliance rate on average was higher among private hospitals than public hospitals (77% vs. 66%); similarly, hospital size correlated to different compliance rates, where large hospitals with a bed capacity of > 200 beds had a higher compliance rate than hospitals with a bed capacity of ≤ 200 beds (81%, 66%, respectively). The eastern region ranked first in compliance with the ESR standards (74%); however, all hospitals had rapid improvement to a great extent.

**Table 2:** Distribution of hospital characteristics by number and percentage of the study population and by compliance rate per year.

Hospital criteria	N	%	Average compliance rate (%)			Average compliance rate (%)
			**2016**	**2017**	**2018**	
**Hospital type “sector”**						
Public	306	70	54.2	69.5	75.7	66
Private	131	30	70.3	77.0	82.6	77
**Hospital size “capacity”**						
≤ 200 beds	337	77	53.8	69.1	75.4	66
> 200 beds	100	23	76.5	80.7	85.8	81
**Hospital located “region”**						
Central	111	25	59.4	75.4	79.9	72
Western	121	28	63.2	70.6	76.8	70
Eastern	71	16	65.7	77.0	78.6	74
Northern	51	12	49.1	62.6	75.0	62
Southern	83	19	53.1	69.9	77.4	67

### Bivariate analysis

The compliance rate was compared by hospital type and bed capacity as follows.

#### ESR compliance rate and hospital type

Independent *t* tests were performed to determine whether hospital type was a factor influencing compliance with ESR standards. Based on the study findings, hospital type proved to be significant, with a P value below 0.05 for nine standards, as shown in Table 3. Overall, private hospitals had a significantly better compliance rate than public hospitals (mean rate = 84% vs. 68%, *t* test = 2.96, P = 0.019).

**Table 3:** Comparison of the mean compliance rate of ESR standards by hospital type (independent *t* test).

Standard	Mean compliance score and SD		Mean difference	*t* test	Sig	95% confidence interval	
	Public hospitals	Private hospitals				Lower	Upper
HR.5	66.63 (13.13)	77.73 (7.70)	− 11.106	− 1.787	0.111	− 25.41	3.2
MS.7	63.79 (19.56)	84.26 (8.9)	− 20.475	− 2.333	0.052	− 41.23	0.28
PC.25	74.11 (11.79)	91.74 (3.04)	− 17.631	− 3.547	**0.013**	− 29.97	− 5.29
PC.26	53.97 (27.7)	69.88 (16.42)	− 15.910	− 1.210	0.260	− 46.14	14.32
QM.17	85.94 (7.75)	92.27 (1.59)	− 6.327	− 1.959	0.103	− 14.44	1.78
QM.18	76.08 (10.59)	93.46 (3.38)	− 17.386	− 3.830	**0.009**	− 28.49	− 6.28
AN.2	72.47 (8.8)	86.76 (4.8)	− 14.287	− 3.491	**0.009**	− 23.78	− 4.79
AN.15	56.67 (13.79)	75.24 (12.68)	− 18.569	− 2.428	**0.036**	− 35.62	− 1.51
IPC.4	85.56 (9.66)	95.36 (2.47)	− 9.806	− 2.409	0.055	− 19.92	0.31
IPC.15	65.67 (15.1)	80.37 (8.84)	− 14.707	− 2.059	0.073	− 31.16	1.74
MM.5	68.11 (12.9)	86.6 (4.44)	− 18.494	− 3.321	**0.015**	− 32.03	− 4.96
MM.6	69.2 (18.1)	87.63 (8.14)	− 18.424	− 2.274	0.057	− 37.62	0.77
MM.41	63.57 (15.79)	86.15 (4.63)	− 22.579	− 3.361	**0.016**	− 39.12	− 6.04
LB.51	56.34 (13.73)	91.59 (8.26)	− 35.246	− 5.388	**0.001**	− 50.27	− 20.22
FMS.9	78.83 (11.87)	92.08 (5.3)	− 13.248	− 2.496	**0.042**	− 25.83	− 0.67
FMS.21	59.89 (11.39)	74.09 (13.86)	− 14.193	− 1.938	0.082	− 30.6	2.21
FMS.22	51.01 (19.54)	71.03 (11.72)	− 20.023	− 2.153	0.063	− 41.39	1.34
FMS.23	76.7 (11.62)	83.44 (6.89)	− 6.740	− 1.222	0.256	− 19.42	5.94
FMS.24	70.09 (8.18)	78.79 (7.09)	− 8.702	− 1.969	0.078	− 18.57	1.17
FMS.32	65.51 (10.82)	80.6 (8.56)	− 15.090	− 2.679	**0.024**	− 27.73	− 2.45
Total Average	68.01 (11.58)	83.95 (6.32)	− 15.947	− 2.962	0.019	− 28.44	− 3.46

Significant values are in bold.

#### Comparing the compliance rate by hospital size “bed capacity”

In comparing compliance rate by bed capacity, although the result of independent *t* test showed eight (8) significant results, the total average of compliance was not significant (P value = 0.119, 95% CI = − 25.9 to 3.5) (Table 4).

**Table 4:** Comparison of the mean compliance rate of ESR standards by hospital size (independent *t* test).

	Bed capacity	Mean	Std. deviation	Independent *t* test	95% CI of the difference	
				Sig. (2-tailed)	Lower	Upper
HR.5	≤ 200 beds	67.1	12.9	0.145	− 24.6	4.3
	> 200 beds	77.3	8.8			
MS.7	≤ 200 beds	67.9	20.3	0.256	− 35.4	10.7
	> 200 beds	80.2	14.5			
PC.25	≤ 200 beds	79.9	14.9	0.424	− 22.5	10.4
	> 200 beds	86.0	9.6			
PC.26	≤ 200 beds	59.2	28.4	0.703	− 37.3	26.2
	> 200 beds	64.7	19.0			
QM.17	≤ 200 beds	86.3	7.7	0.146	− 13.7	2.6
	> 200 beds	91.9	2.8			
QM.18	≤ 200 beds	81.7	13.4	0.397	− 21.5	9.3
	> 200 beds	87.8	10.2			
AN.2	≤ 200 beds	73.9	10.2	**0.048**	− 22.6	− 0.1
	> 200 beds	85.3	6.4			
AN.15	≤ 200 beds	56.6	13.9	**0.034**	− 35.7	− 1.7
	> 200 beds	75.3	12.4			
IPC.4	≤ 200 beds	88.3	10.0	0.412	− 15.5	7.0
	> 200 beds	92.6	6.8			
IPC.15	≤ 200 beds	62.5	11.7	**0.005**	− 33.5	− 8.5
	> 200 beds	83.5	6.0			
MM.5	≤ 200 beds	74.7	16.1	0.519	− 23.3	12.7
	> 200 beds	80.0	10.7			
MM.6	≤ 200 beds	74.7	20.7	0.470	− 29.8	15.1
	> 200 beds	82.1	11.8			
MM.41	≤ 200 beds	71.1	19.4	0.450	− 29.2	14.1
	> 200 beds	78.6	12.9			
LB.51	≤ 200 beds	69.4	21.2	0.489	− 37.1	19.0
	> 200 beds	78.5	22.4			
FMS.9	≤ 200 beds	82.0	13.8	0.310	− 21.8	7.9
	> 200 beds	88.9	7.4			
FMS.21	≤ 200 beds	57.5	10.8	**0.012**	− 32.7	− 5.2
	> 200 beds	76.5	10.5			
FMS.22	≤ 200 beds	47.8	15.1	**0.007**	− 43.5	− 9.3
	> 200 beds	74.2	10.7			
FMS.23	≤ 200 beds	72.4	8.1	**0.005**	− 23.7	− 6.8
	> 200 beds	87.7	2.3			
FMS.24	≤ 200 beds	68.8	7.2	**0.014**	− 19.8	− 2.8
	> 200 beds	80.1	5.9			
FMS.32	≤ 200 beds	65.8	11.2	**0.033**	− 27.5	− 1.4
	> 200 beds	80.3	8.7			
Total average	≤ 200 beds	70.4	13.1	0.119	− 25.9	3.5
	> 200 beds	81.6	8.9			

Significant values are in bold.

#### Comparing compliance rate by hospital type adjusted for year

Using the multivariable analysis (linear regression model), the test was run only on the nine standards that showed a significant result in the bivariate analysis of compliance rate between private and public hospitals: PC.25., AN.15., MM.5., MM.41., LB.51., FMS.9., FMS.32., QM.18., and AN.2.

The results confirmed that the type of hospital has an impact on these nine standards, all of which appear to be statistically significant, as shown in Table 5. Private hospitals had a coefficient of 17.60 (95% CI = 8.8–26.5) and P value < 0.05, indicating more compliance than public hospitals among the 9 standards. Additionally, the compliance rate was found to be significantly higher in standard numbers PC.25, AN.15, MM.5, MM.41, FMS.9, and FMS.32 in 2018 than in 2016. The overall coefficient was 12.84, and the P value < 0.001 for year of compliance (2018 compared with 2016).

**Table 5:** Comparing the compliance rate of the nine significant ESR standards by hospital type adjusted for the year.

Hospital characteristics	Coefficient	P value	95% Confidence interval	
			Lower bound	Upper bound
**PC.25**				
Intercept	− 11,805.6	0.038	− 22,758.7	− 852.4
**Hospital type**				
Public	1.00 (ref)			
Private	17.60	**0.002**	8.8	26.5
**Year**				
2016	1.00 (ref)			
2017	9.84	0.076	− 1.34	21.02
2018	11.75	**0.042**	0.57	22.93
**QM.18**				
Intercept	− 10,362.5	0.043	− 20,326.3	− 398.7
**Hospital type**				
Public	1.00 (ref)			
Private	17.4	**0.001**	9.3	25.5
**Year**				
2016	1.00 (ref)			
2017	5.34	0.282	− 5.49	16.16
2018	10.32	0.059	− 0.5	21.15
**AN.2**				
Intercept	− 2502.3	0.354	− 8369.4	3364.9
**Hospital type**				
Public	1.00 (ref)			
Private	14.3	**< 0.001**	9.5	19
**Year**				
2016	1.00 (ref)			
2017	2.54	0.368	− 3.7	8.78
2018	2.52	0.371	− 3.72	8.761
**AN.15**				
Intercept	− 15,602.6	0.003	− 24,285.4	− 6919.7
**Hospital type**				
Public	1.00 (ref)			
Private	18.7	**< 0.001**	11.7	25.8
**Year**				
2016	1.00 (ref)			
2017	7.88	0.089	− 1.55	17.32
2018	15.48	**0.006**	6.04	24.92
**MM.5**				
Intercept	− 16,939.2	0.004	− 26,648.1	− 7230.2
**Hospital type**				
Public	1.00 (ref)			
Private	18.5	**0.001**	10.6	26.4
**Year**				
2016	1.00 (ref)			
2017	11.70	0.028	1.7	21.69
2018	16.84	**0.005**	6.84	26.83
**MM.41**				
Intercept	− 18,654.3	0.011	− 31,618.6	− 5690
**Hospital type**				
Public	1.00 (ref)			
Private	22.6	**0.001**	12.1	33.1
**Year**				
2016	1.00 (ref)			
2017	12.24	0.073	− 1.51	25.99
2018	18.53	**0.015**	4.77	32.28
**LB.51**				
Intercept	2142.4	0.798	− 16,510.4	20,795.3
**Hospital type**				
Public	1.00 (ref)			
Private	35.2	**0.001**	20.1	50.3
**Year**				
2016	1.00 (ref)			
2017	− 13.09	0.095	− 29.12	2.93
2018	− 2.12	0.764	− 18.14	13.91
**FMS.9**				
Intercept	− 14,357.8	0.01	− 24,281.1	− 4434.4
**Hospital type**				
Public	1.00 (ref)			
Private	13.2	**0.005**	5.2	21.3
**Year**				
2016	1.00 (ref)			
2017	9.97	0.057	− 0.41	20.36
2018	14.29	**0.014**	3.91	24.68
**FMS.32**				
Intercept	− 8752.6	0.031	− 16,450.7	− 1054.5
**Hospital type**				
Public	1.00 (ref)			
Private	15.1	**0.001**	8.9	21.3
**Year**				
2016	1.00 (ref)			
2017	0.39	0.902	− 6.91	7.69
2018	8.71	**0.026**	1.41	16.01
**Total average**				
Intercept	− 12,918.9	0.004	− 20,379.1	− 5458.6
**Hospital type**				
Public	1.00 (ref)			
Private	15.9	**0.001**	9.9	22
**Year**				
2016	1.00 (ref)			
2017	7.61	0.060	− 0.41	15.62
2018	12.84	**0.007**	4.82	20.86

Significant values are in bold.

## Discussion

This is the first study to focus on ESR standards as an indicator of accreditation effectiveness, which may give professionals who are interested in this area an overview of the accreditation output and whether they are on the right track or need to take further corrective actions. This study found that there was a significant increase in the compliance rate that was considered a positive indication of increasing patient safety, i.e., if the compliance rate increases, the implementation of safety requirements is increased, which may lead to an increased patient safety level. In total, hospital type was the most significant factor influencing the compliance rate, and private hospitals had a significantly higher compliance rate than public hospitals.

There is a general increase in the compliance rate by year, probably because hospitals become more familiar with the process and understand it better; they are aware of standards and how to apply them properly, especially paper-work requirements (apart from the fact that most hospitals now have one or more of their employees working as a CBAHI surveyor, who could play the role of an internal consultant to his or her hospital). It was demonstrated that a higher rate of adoption of standards related to adequate and reliable information received on the standards when authorities engaged in adoption of these standards^4^. Most of the ESR standards have assumed a rising trend from the first survey in 2016 to the last in 2018, especially in seven of these standards, which have the highest difference in compliance rate. Especially in standard PC.26 (identify and manage patients at risk of developing venous thromboembolism (VTE)), where the average variance in compliance rate has increased by 56% in three years, the compliance rate has increased significantly from 2016 to 2018. Initially, it was an initiative of the Ministry of Health who adopted the program to increase recognition of the impact of VTE and raise the level of patient safety. Therefore, it was shown that proper awareness of accreditation elements such as workshops increases the level of compliance among hospital workers^6^.

The study showed that hospitals with larger bed capacity had a higher compliance rate than smaller hospitals. Although a limited number of studies have assessed the relation between accreditation and hospital size, a high level of compliance is seen among larger hospitals, usually including teaching hospitals, that are more likely to adopt electronic medical records that improve hospital quality than small hospitals^7^. These results, however, are in contrast with the study by Ardalan et al*.,* where hospitals with small hospital sizes with ≤ 100 beds showed higher levels of total safety than hospitals with capacities greater than 100 beds^8^.

The results of this study showed that the Northern region had the highest variance rate (26%) in compliance from the first ESR survey in 2016 compared with the latest survey in 2018; however, the Central region topped the highest compliance rate in 2018 by 80%. The study results by Almalki, FitzGerald and Clark^9^ indicated that there is also a difference between the performance of hospitals from different regions in the Kingdom, but there are no studies looking at the reasons why the Kingdom's regions differ in their adherence to accreditation requirements. However, we believe it is likely that some areas—such as the South region and some of the remote or rural areas in the Kingdom—lack the basic elements of modern lifestyle that are available in urban areas and large developed cities^9^; therefore, most health professionals avoid working in these areas, clustering progress in resources and employment in major cities such as Riyadh, Jeddah and Dammam. Thus, progress in applying quality and patient safety standards is concentrated in the Central, Eastern and Western regions. Another study agreed with this reasoning, where he mentioned in his study that the private sector in recent years is moving to expand its services and focus on large cities^10^.

The current study showed a higher compliance rate in private hospitals (11%) than in government hospitals. This variance is due to several reasons. First, since the renewal of the private hospital license is currently linked to the passage of accreditation, the owners of private hospitals fear the closing their business, so they become keen to commit to passing the accreditation in a high score; thus, they do not lose their customers, who in this case are the patients, raising the level of public awareness of health and encouraging the patients to look for certified hospitals^11^. Second, the budget for governmental hospitals plays a role in applying the necessary changes in facility structure, where these budgets often come from their governing bodies in limited and fixed amounts at the beginning of the year^10^, which would delay the implementation of safety standards. In contrast, private hospitals do not have a limited budget, such as most government hospitals, especially military hospitals, thus making it easier for them to apply requirements related to the safety of the facility and keen to build hospitals correctly and, in a manner, consistent with standards from the beginning^11^. Budget concerns also contribute to enabling private hospitals to bring the latest technologies and thus save them time, reduce errors and make them more professional and more compliant with the standards^10^. A study showed that a decrease in crude oil revenue worldwide in 2015 led to a significant decrease in governmental funds allocated to the Ministry of Health (MOH) budget in 2016 (34.5% less than 2015); perhaps this is one of the reasons that affected the rate of compliance of MOH hospitals at the time^12,13^. Third, in private hospitals, staff-quality leaders in particular are afraid of losing their jobs in case of poor results or failure to pass the survey^14^. Fourth, from a materialistic perspective, the increased losses due to medical errors are more expensive than the cost of prevention.

The government should also support the implementation of the National Transformation Program (Saudi Vision, 2030) goals in raising the level of health services by extending the responsibility of applying the accreditation to all involved and seeking the collaboration of the important stakeholders (i.e., other government agencies and relevant bodies such as the Ministry of Labor, Saudi Consuls, Civil Defense, electricity, water, pharmaceutical and medical gas companies, and blood donation charities) to get their buy-in for supporting the achievement of this goal. As the results showed that private hospitals are more compliant than government hospitals in applying standards, and as we are heading toward privatization^9,12^, this is a good indicator of future direction, where superiors and investors in government hospitals should focus on benefiting from the experience of private hospitals in the accreditation process. Additionally, infrastructure should be focused on in the establishment of any new hospital to consider facility management and safety requirements from the beginning^15^.

The study has several limitations. As a cross-sectional study, causality cannot be determined, and we cannot discard the possibility that our results may be confounded by the increased knowledge of standards and how they are applied. We cannot be certain that the level of patient safety was low and increased due to accreditation impact. It is possible that the increase was in the application of requirements not in the actual level of patient safety, which could create a bias toward finding the benefit of accreditation. Given that the mandatory application of ESR standards has been introduced recently and that there have been an insufficient number of studies on the impact of accreditation on patient safety in the KSA (nor CBAHI or ESR), we lack sufficient evidence that would confirm the impact of accreditation with certainty. Finally, the dates of surveyors’ visits were announced in advance, and the hospitals were notified before the visit in sufficient time to prepare for the survey, which may affect the credibility of the results. However, this behavior changed in 2019, as the CBAHI that visits would be unannounced.

One of the major strengths of this study is that we used a national database that includes all surveyed hospitals across the Kingdom of Saudi Arabia, and the risk of information or selection bias was minimized. Although some hospitals were excluded from the analysis due to ineligibility, those excluded samples did not exceed 10%, which is within the acceptable limit for data exclusion.

## Conclusion

This study is one of the first national studies to assess ESR compliance. This assessment revealed that using ESR as a preliminary requirement before CBAHI accreditation is practical and could help to identify the weak areas that need improvement. The average compliance rate with ESR is increasing each year as compliance with ESR standards has become mandatory. Compliance with quality standards is challenging and requires more collaborative effort between hospital leaders and health care staff to identify the gaps for improvement and focus their improvement efforts.

This study supports mandatory accreditation programs for both public and private health sectors, with increased monitoring by the concerned parties (i.e., CBAHI and Ministry of Health). Governing bodies should also ensure that their affiliated hospitals comply with these standards, which proved to be in the interests of the patient and the hospital-provided services.

## Data Availability

The data that support the findings of this study are available from the corresponding author upon reasonable request.
